# Circadian rhythm dysfunction and clinical heterogeneity in pediatric ADHD: A critical need for innovation in assessment and treatment

**DOI:** 10.1002/jcv2.12281

**Published:** 2024-08-27

**Authors:** Jessica R. Lunsford‐Avery, J. Lynn Davis, Michael T. Willoughby

**Affiliations:** ^1^ Duke University School of Medicine Durham North Carolina USA; ^2^ RTI International Research Triangle Park North Carolina USA

**Keywords:** ADHD, circadian rhythm, sleep

## Abstract

Despite success of pharmacologic and psychosocial treatments, attention‐deficit/hyperactivity disorder (ADHD) frequently results in significant personal impairment and socioeconomic burden. A challenge for the development of effective treatments targeting functional deficits in ADHD is the substantial heterogeneity in clinical features such as comorbidity, executive dysfunction, and cognitive disengagement. In this editorial perspective, we consider circadian dysfunction as a potentially critical mechanism underlying clinical heterogeneity and discuss current barriers to identifying and treating circadian dysfunction in pediatric ADHD. Recent advances in wearable sensors may offer new opportunities to elucidate the underlying role of circadian dysfunction in ADHD heterogeneity and support the development of personalized sleep treatments with the power to improve long‐term educational, interpersonal, and occupational outcomes for children with ADHD.

**Key points**

- A challenge for the development of effective treatments targeting functional deficits in attention‐deficit/hyperactivity disorder (ADHD) is the substantial heterogeneity in clinical features.
- Sleep disturbances of both behavioral and biological origin are common in ADHD and may underlie clinical heterogeneity.
- Identification of biological (circadian) dysfunction in pediatric ADHD is hampered by the burden of gold standard circadian assessments such as dim light melatonin onset.
- Advances in wearable sensors offer new opportunities for rigorously testing the potential role of circadian dysfunction in contributing to clinical heterogeneity in pediatric ADHD and for developing personalized ADHD treatments.

Attention deficit/hyperactivity disorder (ADHD) is a common, persistent psychiatric condition of childhood. Despite the success of pharmacologic and psychosocial treatments, ADHD results in significant personal impairment, distress, and socioeconomic burden to families and society (Doshi et al., 2012). For example, ADHD is associated with impaired interpersonal relationships, educational and employment under‐attainment, greater health problems, and earlier mortality (Cherkasova et al., 2021). Optimal treatments for ADHD must go beyond symptom management to ameliorate social, educational, and occupational impairment and improve quality of life (Rostain et al., 2015).

A key barrier to developing effective treatments targeting functional deficits is the marked clinical heterogeneity within the ADHD population (Luo et al., 2019). In addition to variability in pharmacologic and psychosocial treatment response, ADHD is characterized by heterogeneity in co‐occurrence of internalizing and externalizing conditions, executive functioning deficits, and cognitive disengagement syndrome. When present, these clinical features independently predict increased functional deficits and poorer prognosis in ADHD (Burns & Becker, 2021; Luo et al., 2019). However, the biological mechanisms that underlie heterogeneity in clinical presentation and treatment response are poorly understood. Identifying and targeting processes underlying ADHD heterogeneity early in life is essential for improving long‐term clinical outcomes.

Sleep disturbances are key candidates for clarifying clinical heterogeneity and personalizing ADHD treatment. Sleep problems are highly prevalent among children with ADHD, including greater bedtime resistance and sleep onset latency (SOL), difficulties falling asleep, and delayed and irregular sleep patterns (Bondopadhyay et al., 2022). Sleep disturbances increase ADHD severity, persist despite best‐practice ADHD treatment, and may be further compounded by stimulant medication, inconsistent use of immediate release melatonin, and caffeine use. Two distinct mechanistic pathways may underlie sleep problems in ADHD (Gruber et al., 2012). In the behavioral pathway, longer SOL and delayed/irregular sleep patterns are manifestations of ADHD symptoms (e.g., disorganization) and comorbidity (e.g., oppositionality), which result in bedtime resistance. In the biological pathway, sleep complaints arise from an endogenous delay in the circadian rhythm. That is, a “mismatch” exists between a child's biological clock, as determined by clock gene expression interacting with external cues (e.g., light exposure), and their social clock setting daily schedules (e.g., school, family routines).

Although both pathways result in reduced sleep quality and daytime sleepiness, they represent discrete mechanistic processes that may benefit from distinct treatments (Gruber et al., 2012). Research traditionally has focused on the behavioral pathway, which is assessed with minimally‐invasive methods (i.e., parent report) and treated via behavioral sleep therapy. Behavioral sleep treatments improve sleep and core ADHD symptoms as well as psychosocial and executive functioning in children with ADHD (Bondopadhyay et al., 2022). However, behavioral sleep treatments are not effective for all children with ADHD and sleep concerns. Among children with ADHD whose sleep problems result from an endogeneous circadian delay, chronotherapies may be warranted (Van der Heijden et al., 2005). In addition, there is likely a subset of children with ADHD who experience sleep disturbances stemming from a combination of these behavioral, biological, and environmental processes. A primary barrier to developing personalized sleep medicine approaches in pediatric ADHD is a lack of accessible methods to distinguish children whose sleep problems reflect behavioral versus biological pathways, or a combination of these mechanisms.

Important to the development of personalized treatments is that delayed circadian phase is linked to the clinical features that are common, but not universal, in ADHD, and thus may represent a key biological mechanism underlying clinical heterogeneity. Regarding comorbidity, while co‐occurring behaviors such as oppositionality may increase behavioral sleep problems, delayed circadian phase is also increasingly recognized as a transdiagnostic risk factor for onset of internalizing and externalizing behaviors in children (Arns et al., 2021). Delayed circadian phase, and its impact on restricting sleep duration (due to the mismatch with social schedules), is also a risk factor for executive functioning, working memory, and inhibitory control deficits (Taillard et al., 2021). Finally, sleep problems often related to circadian delay, including longer SOL, difficulty falling asleep, later sleep onset, and eveningness chronotype, are related to cognitive disengagement in youth (Fredrick et al., 2022). Within ADHD specifically, general sleep disturbances are clearly related to these clinical features. Disturbed sleep precedes, predicts, and contributes to internalizing and externalizing behaviors among youth with ADHD (Dimakos et al., 2021), and sleep and cognition in ADHD are tightly linked (Bondopadhyay et al., 2022). However, the specific role of biological versus behavioral sleep problems in determining comorbidity, cognitive deficits, and cognitive disengagement within ADHD is currently unclear.

Studies suggest a high prevalence of circadian delay in adults with ADHD based on behavioral, endocrine, and genetic indicators, which in turn increases ADHD‐related morbidity (Bijlenga et al., 2019). These findings align with longstanding theoretical models of ADHD pathogenesis implicating atypical arousal systems (Sergeant, 2000). Mechanisms linking ADHD and circadian delay likely involve overlap in neuroanatomy, genetics, and the role of light exposure in circadian system regulation, arousal, and attention (Bijlenga et al., 2019). Geographic areas with higher solar intensity levels have lower ADHD prevalence, providing indirect support for a role of circadian dysfunction in ADHD etiology (Arns et al., 2013). Increased solar intensity may align a person's biological and social clocks, reducing circadian contributions to ADHD symptoms.

In contrast, little is known about circadian function and ADHD in childhood, the peak period of ADHD referral and the developmental stage by which circadian preference manifests (evening “owls” vs. morning “larks”), which is largely genetically‐determined and a stable trait into adulthood (Merikanto et al., 2018). A recent review cited only 11 studies (Bondopadhyay et al., 2022), and only one study using the gold‐standard circadian assessment, dim light melatonin onset (DLMO) (Van der Heijden et al., 2005). Initial support for circadian delay in children with ADHD includes lower salivary cortisol awakening response, lower salivary cortisol across the day, higher heart rate in the afternoon/evening, and shortened nocturnal melatonin signal compared to healthy controls, as well as associations between eveningness chronotype and sleep problems among children with ADHD (Bondopadhyay et al., 2022).

Findings to date, however, are mixed and subject to methodological limitations (Bondopadhyay et al., 2022). Although daytime diurnal cortisol slope, cortisol awakening response, and heart rate have circadian influence, they are not direct measures of the circadian clock with respect to habitual bedtime. Similarly, although eveningness and delayed sleep patterns are behavioral proxies for delayed circadian phase, they are inconsistently correlated with DLMO in children (Reiter et al., 2020), and may be influenced by non‐circadian‐related characteristics of the child (e.g., bedtime resistance) or family (e.g., unstable bedtime routines). Finally, prior studies using small samples and case‐control designs are unable to detect whether circadian dysfunction characterizes a subset of children within the ADHD population most likely to benefit from chronotherapies.

Given the literature implicating circadian dysfunction in adult ADHD (Bijlenga et al., 2019), and the stability of circadian preference from childhood to adulthood (Merikanto et al., 2018), it is perhaps surprising that there are so few studies of circadian function, and its relationships with clinical features, among school‐aged children during the peak period of ADHD referral and diagnosis. This dearth of research, and subsequent lack of application in clinical settings, is likely due to the difficulty of directly measuring circadian functions in children, especially those with behavioral challenges (Bondopadhyay et al., 2022). Gold‐standard circadian measurement (DLMO) requires repeated biospecimen collection in controlled light settings over multiple hours (Reid, 2019). As a result, it is burdensome to families and infeasible for routine diagnostic intakes. Thus, minimally‐intrusive circadian assessments in children's natural environments are critically needed.

Advances in wearable sensors, particularly those assessing core body temperature (CBT), as well as novel metrics from existing rest/activity sensors, offer new opportunities to identify children with circadian dysfunction. While DLMO is more highly correlated to sleep timing, CBT values such as the temperature nadir are also correlated to sleep timing and can serve as proxies for melatonin production (Zulley et al., 1981). CBT peaks in the evening, decreases with sleep onset, reaches a nadir in the late evening/early morning, and increases in the hours prior to waking. CBT was traditionally measured using invasive methods such as ingestible “ePill” thermometers (Reid, 2019). However, innovations in wearable heat flux sensors provide new opportunities to measure CBT noninvasively.

Heat flux sensors measure time‐locked changes in CBT expressed as body heat through the skin. In the absence of heat loss to the environment, the skin temperature equilibrates to CBT. Recent approaches, which simultaneously measure skin temperature and heat flux, permit CBT estimation using computational methods and have accurately estimated circadian rhythm with promising initial results (Mendt et al., 2017). Wearable heat flux sensors permit noninvasive, continuous CBT monitoring under free‐living conditions. In prior work with heat flux sensors, circadian parameters (e.g., mesor, amplitude, acrophase) did not significantly differ from those computed from a rectal probe (Gunga et al., 2009). In addition to heat flux sensors, new metrics derived from wearable rest‐activity/actigraphy sensors (i.e., both rest/activity and heart rate [and heart rate variability]) may be suitable proxies for circadian rhythm in children in addition to providing important information about sleep (e.g., sleep regularity, latency, continuity) (Moreno et al., 2022).

Thus, innovations in wearable heat flux sensors and in the extraction of new metrics from rest/activity sensors may provide novel opportunities for objective, holistic sleep and circadian assessments in children with ADHD in home settings. Rigorous studies using gold‐standard circadian assessment (i.e., DLMO) and wearable sensors are essential to clarify the prevalence and impact of sleep disturbances of circadian origin in pediatric ADHD, and whether sensor data can be effectively used as a DLMO proxy in this population. If wearable sensors can identify children with circadian dysfunction, pediatric primary care clinics might incorporate sensor‐based, in‐home sleep evaluations into routine clinical assessment for children who present with ADHD concerns. Such evaluations would serve to identify a specific biological process which may underlie clinical outcomes and provide a method for measuring the effectiveness of treatments that alleviate symptoms of the biological pathway.

Accurate identification of circadian dysfunction in pediatric ADHD may also facilitate innovations in personalized treatments. For example, future studies may investigate blue light therapy as an augment to existing empirically‐supported best practices as a means for improving ADHD symptoms and related clinical features (e.g., comorbidity, executive function, cognitive disengagement). Exposure to specific wavelengths of light (e.g., blue light at 480 nm) at certain times of the day induces activity in a nonvisual retinal pathway that projects to the areas of the brain that control the master circadian clock and foster alertness and information processing (Lucas et al., 2014). Since the spectral composition and temporal nature of light is known to impact circadian phase, customized phototherapy approaches may ultimately be possible.

Effectively targeting functional impairments and quality of life in ADHD requires a new approach personalized to the biology and behavior of each child. Given both the prevalence of sleep disturbances in pediatric ADHD and the evidence for circadian delay in affected adults, developing accessible, accurate, and scalable measures of circadian function must be a clinical priority. Innovations in the deployment and analysis of wearable sensors provide exciting new opportunities for rigorously testing the potential role of circadian dysfunction in contributing to clinical heterogeneity in pediatric ADHD and for developing personalized sleep treatments with the power to improve educational, interpersonal, and occupational outcomes across the lifespan.

## AUTHOR CONTRIBUTIONS

**Jessica R. Lunsford‐Avery**: Conceptualization; methodology; writing—original draft; writing—review & editing. **J. Lynn Davis**: Conceptualization; methodology; writing—review & editing. **Michael T. Willoughby**: Conceptualization; methodology; writing—review & editing.

## CONFLICT OF INTEREST STATEMENT

The authors have declared no competing or potential conflicts of interest.

## ETHICAL CONSIDERATIONS

Ethical approval was not required for this study.

## Data Availability

Data sharing is not applicable to this article as no new data were created or analyzed in this study.
